# Effect of Resin Cement Type and Thickness on Color Stability and Cement Translucency: An In-vitro Study -

**DOI:** 10.31661/gmj.v14iSP1.4011

**Published:** 2025-12-15

**Authors:** Mahmoud Sabouhi, Babak Naziri, Berahman Sabzevari, Farshad Bajoghli, Mohammadhossein Fakoor, Sarah Noorizadeh

**Affiliations:** ^1^ Department of Prosthodontics, Dental Materials Research Center, Dental Research Institute, School of Dentistry, Isfahan University of Medical Sciences, Isfahan, Iran; ^2^ Department of Prosthodontics, School of Dentistry, Islamic Azad University, Tehran Branch, Tehran, Iran; ^3^ Orthodontist, Private Practice, Mashhad, Iran; ^4^ Dental Implants Research Center, Department of Prosthodontics, School of Dentistry, Isfahan University of Medical Sciences, Isfahan, Iran; ^5^ Department of Restorative Dentistry, School of Dentistry, Tehran University of Medical Sciences, Tehran, Iran; ^6^ Department of Periodontics, School of Dentistry, Shahid Beheshti University of Medical Sciences, Tehran, Iran

**Keywords:** Resin Cement, Light-cure, Dual-cure, Color Stability, Translucency, Cement Thickness

## Abstract

**Background:**

It is widely accepted that discoloration of resin cements is a common problem, especially in translucent restorations, which causes discoloration of restoration and the need for replacement. The purpose of this study was to investigate the effect of resin cement type and thickness on color stability and cement translucency.

**Materials and Methods:**

The present in vitro study was conducted on 120 disc-shaped 5-mm samples in three thicknesses of 50, 100 and 150 microns within 12 groups using two light-cure resin cements (ultra-Bond and option 2) and two amine-free dual-cure resin cements (V5 and NX3) by silicone mold. All specimens were thermocycled under 8000 rpm and then the mean color and translucency were determined for all specimens. Data were analyzed using SPSS software.

**Results:**

Thermal cycle at 8000 rpm significantly increased color change (ΔE) and decreased translucency parameter (TP) in all four resin cements (P0.001), although ΔE was clinically acceptable for all cements. (ΔE≤3.3). In addition, increased cement thickness caused an increase in ΔE and a decrease in translucency changes (ΔTP) (P0.001).

**Conclusion:**

The resin cement type and thickness had an effect on color stability and cement translucency. The light-cure cements and new dual-cure amine-free cements had clinically acceptable color stability.

## Introduction

Tooth-colored restorations are now widely used to meet the esthetic needs of patients [1][2]. However, the color matching of tooth-colored restorations such as all-ceramic crowns, porcelain laminate veneers, ceramic inlays, and onlays with natural teeth has always been a challenge [3]. The final color of a ceramic restoration is determined by a combination of factors such as the shade of the tooth or underlying restoration, and the color and thickness of the cement and ceramic [4]. Discoloration of resin cements is a common problem, especially in translucent restorations, which leads to discoloration of the restoration and the need for replacement [1][2][5].

Based on polymerization type, resin cements are classified into three categories: self-cured (chemically cured), light-cured, and dual-cured [6][7][8][9][10]. Recently, dual-cure amine-free cements without benzoyl peroxide initiators have been introduced. These amine-free resin cements have advantages such as fluoride release, strong covalent bonding, ease of use, improved color stability, and enhanced esthetics [1][11].

Clinical evaluation of color change has been the subject of many studies. The CIE (Commission Internationale de l'Eclairage) system has been proposed to measure color change (ΔE) based on the color parameters Lab*. The ΔE value is used to determine whether overall color change is perceptible to the observer. The magnitude of color difference (ΔE) reflects human perception of color, representing the numerical distance between the Lab* coordinates of two colors [3][12][13].

In addition to color stability, translucency is also an important factor in the selection of resin cement.

A reduction or increase in cement translucency changes the appearance of the underlying color and, over time, affects the esthetics of the restoration [14]. Resin cement translucency is usually evaluated using the translucency parameter (TP) or contrast ratio (CR). In recent studies, TP has been used to measure changes in resin cement translucency, although a clinically acceptable limit for TP has not been reported [5][14][15].

Resin cement thickness is one of the most influential factors affecting the final color and esthetics of ceramic restorations. In cases where the restoration substructure has an undesirable color due to tooth discoloration or the use of metal-colored infrastructures, such as base metal custom posts, the thickness and color of the porcelain and cement are important in determining the final shade of the restoration [6].

Ghavam M. et al. reported that light-cure and dual-cure resin cements showed discoloration after aging, although the changes were clinically acceptable. In terms of translucency, they concluded that autopolymerized cements showed significant increases in opacity [16]. Hao Yu et al. also reported higher color stability and translucency in light-cure resin cements compared to dual-cure ones [5].

Other studies have shown that aging results in clinically unacceptable discoloration and translucency changes in resin cements [17]. Cagri Ural et al. [1] concluded that both chemical composition and curing method affect color stability and translucency.

Most previous studies have focused on external factors affecting the color stability and translucency of resin cements (such as colored drinks, food, and smoking), while few have investigated the role of cement composition. The development of new amine-free resin cements highlights the need to evaluate how their ingredients affect color stability and translucency. Moreover, considering the increasing use of tooth-colored resin-bonded restorations, the clinical importance of color stability and translucency, and the introduction of new amine-free dual-cure resin cements with limited related studies, further research in this area is necessary.

The purpose of this study was to investigate the effect of resin cement type and thickness (light-cure and amine-free dual-cure) on color stability and cement translucency.

## Materials and Methods

The present in vitro fundamental and applied research was conducted at the Professor TorabiNejad Dental Research Center, Isfahan, Iran, in February 2019. The study evaluated two light-cure resin cements (Ultra-Bond and Option 2) and two amine-free dual-cure resin cements (V5 and NX3), with their compositions detailed in Table-1.

### Sample Selection Criteria

Inclusion criteria included amine-free dual-cure and light-cure resin cement disks with specific dimensions and thicknesses, free from fractures or cracks. Exclusion criteria eliminated any sample that exhibited fractures or cracks during preparation. All cements were selected in the same shade and clear color to eliminate the influence of color shade on results [14].

### Sample Preparation

Initially, 120 disc-shaped samples, each with a 5-mm diameter, were prepared in three thicknesses: 50, 100, and 150 microns using silicone molds. For each resin cement and thickness, 10 disc-shaped samples were created, resulting in a total of 12 groups (10 samples per cement per thickness). To ensure sufficient thickness for polishing, an additional 0.02 mm was incorporated into the mold design. The resin cement was injected into the mold, covered with a glass plate and Mylar tape, and excess cement was removed by applying finger pressure from the mold to the glass plate [5].

### Curing Process

The resin cements in all groups were cured using a light-curing device (Valo-cordless; Ultradent Products, South Jordan, Utah) for 40 seconds at an intensity of 1000 mW/cm². After curing, samples were removed from the silicone molds and polished using a series of silicon carbide sheets (600, 800, 1000, and 1500 grits). The final thicknesses were adjusted to 50 ± 5, 100 ± 5, and 150 ± 5 microns, verified with a digital micrometer (IP-65 Digimatic Micrometer; Mitutoyo, Japan) [1].

### Pre-measurement Preparation

Prior to color measurement, all specimens were cleaned in an ultrasonic cleaner with distilled water for 10 minutes and dried using oil-free compressed air for 30 seconds [5].

### Color Measurement

The color of each specimen was measured in triplicate using a spectrophotometer (Vita Easyshade V, Zahnfabrik, Germany) at the sample’s midpoint, with the mean calculated to establish the baseline color based on the CIE Lab system*. This system evaluates color in a uniform three-dimensional space, capturing L (brightness), a (red-green), and b (yellow-blue)* parameters via reflected wavelength or light. A custom silicone mold was used to position specimens in the spectrophotometer, shielding them from ambient light. The device was calibrated before each measurement per the manufacturer’s instructions [1][5].

### Aging Simulation

All specimens underwent thermocycling at 5°C and 55°C with an immersion time of 15 seconds and a waiting time of 3 seconds at 8000 rpm, simulating one year of clinical use [18]. Post-thermocycling, the mean Lab* parameters were recalculated using the spectrophotometer. The ΔE value, indicating color difference, was calculated for each sample after aging using the equation:


\Delta E = \sqrt{(L_{1} - L_{2})^{2} + (a_{1} - a_{2})^{2} + (b_{1} - b_{2})^{2}}

Where L represents brightness, a denotes the green-red color ratio, and b indicates the yellow-blue color ratio. Subscripts 1 and 2 correspond to color parameters before and after thermocycling, respectively. A higher ΔE value signifies a greater color difference [5][13].

### Translucency Evaluation

All specimens were evaluated by the spectrophotometer before and after aging, with measurements taken three times on a white background and three times on a black background. The mean Lab* parameters were recorded for both backgrounds, and the TP (translucency parameter) index was calculated before and after aging using the following equation:


\Delta E = \sqrt{(L_b^{\circ} - L_w^{\circ})^{2} + (a_b^{\circ} - a_w^{\circ})^{2} + (b_b^{\circ} - b_w^{\circ})^{2}}

Where W represents color coordinates on the white background, and B denotes data from the black background. Larger TP values indicate greater translucency [14][15].

### Statistical Analysis

All statistical analyses were performed using SPSS software version 22 (SPSS, Inc., Chicago, IL). One-way ANOVA and Tukey’s post hoc tests were used to analyze ΔE values, while the Kruskal-Wallis test and Bonferroni post hoc test were employed to assess ΔTP values.

## Results

**Table:** Table 2. Mean and Standard Deviation of Color Difference in Three Thicknesses of 50, 100, and 150 Microns after Thermocycling

Cement type	Thickneses (µ)	Mean	SD	SE	**95% Confidence Interval for Mean **	
					Lower Bound	Upper Bound
	50	0.84	0.0170	0.0033	0.83	0.85
Choice 2	100	0.95	0.0176	0.0042	0.94	0.96
	150	1.06	0.0165	0.0041	1.05	1.07
	50	0.90	0.0154	0.0036	0.89	0.91
Ultra bond	100	1.00	0.0096	0.0032	1.00	1.01
	150	1.12	0.0095	0.0056	1.11	1.12
	50	1.82	0.0167	0.0065	1.81	1.83
Nx3	100	2.04	0.0173	0.0025	2.03	2.06
	150	2.24	0.0168	0.0036	2.23	2.25
	50	1.63	0.0175	0.0033	1.62	1.64
Panavia v5	100	1.80	0.0182	0.0031	1.79	1.81
	150	2.03	0.0169	0.0037	2.02	2.04

**Table:** Table 3. One-way ANOVA Test Results for Discoloration

	Sum of Squares	df	Mean Square	F	Sig.
Between Groups	29.947	11	2.722	11894.064	.000
Within Groups	.025	108	.000		
Total	29.971	119			

**Table:** Table 4. Mean and Standard Deviation of Translucency before (TP1) and after (TP2) Thermocycling and Translucency Change (ΔTP) in Three Thicknesses of 50, 100, and 150 Microns

Cement type	Thickneses (µ)	TP1	TP2	abs ΔTP
	50	12.08±0.01	11.65±0.02	0.42±0.01
Choice 2	100	11.94±0.01	11.41±0.01	0.52±0.01
	150	11.76±0.01	11.15±0.01	0.61±0.00
	50	11.96±0.01	11.49±0.01	0.46±0.00
Ultra bond	100	11.82±0.01	11.26±0.01	0.55±0.01	
	150	11.66±0.01	11.03±0.01	0.63±0.00
	50	11.67±0.02	11.32±0.01	0.34±0.01
Nx3	100	11.53±0.01	11.08±0.01	0.44±0.01
	150	11.38±0.01	10.87±0.01	0.50±0.01
	50	11.71±0.01	11.41±0.00	0.30±0.01
Panavia v5	100	11.56±0.01	11.17±0.01	0.39±0.01
	150	11.41±0.01	10.95±0.01	0.46±0.01	

**Figure-1:**
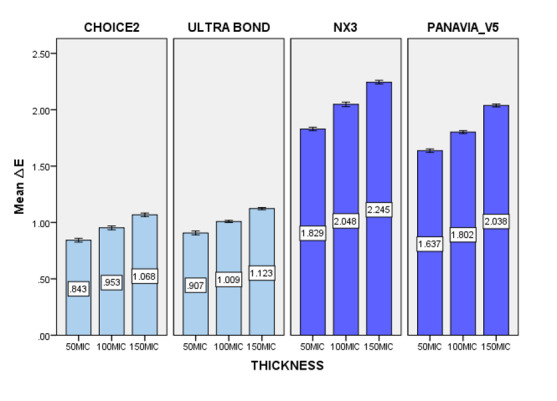
Figure 1. Mean discoloration (ΔE) of resin cements in three thicknesses of 50, 100 and 150 microns after thermocycling

**Figure-2:**
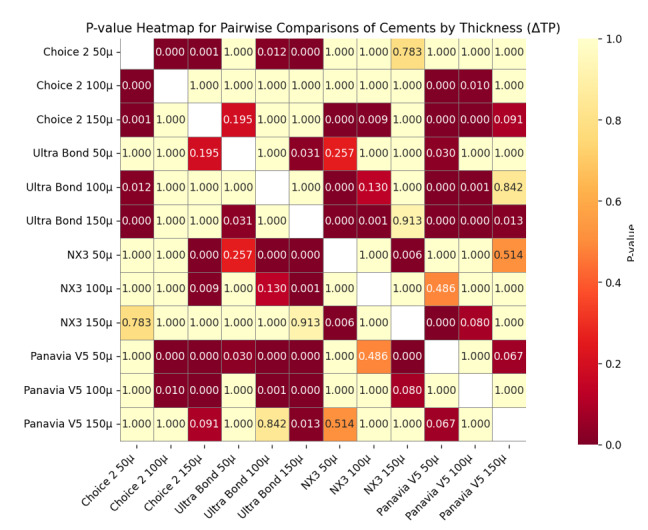
Figure 2. P-value Heatmap for Pairwise Comparisons of Cements by Thickness (ΔTP)

**Figure-3:**
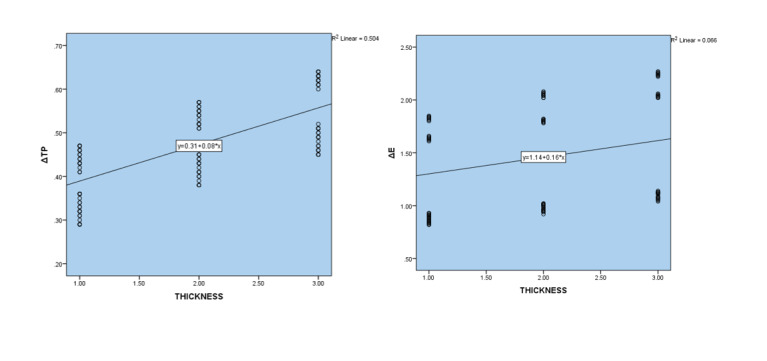
Figure 3. Correlation of thickness changes from 50 to 150 microns with discoloration (left graph) and translucency changes (right graph)

### Assessment of Discoloration (ΔE)

The mean ΔE values and standard deviations for all resin cement samples are summarized in Table-2. The highest ΔE value was observed for dual-cure NX3 cement at a thickness of 150 µm (2.24 ± 0.01), indicating the greatest color change after thermocycling. In contrast, the lowest ΔE value was recorded for Choice 2 cement at 50 µm (0.84 ± 0.01), reflecting minimal discoloration (Figure-1). The results of the one-way ANOVA (Table-3) revealed significant differences among the cement groups (P<0.001). Tukey’s post hoc test indicated that all groups differed significantly from each other (P<0.001), except for the NX3 cement at 100 µm and Panavia v5 cement at 150 µm, which did not differ significantly (P=0.943).

### Assessment of Translucency Changes

The mean translucency indices before (TP1) and after (TP2) thermocycling, as well as the absolute changes (ΔTP) and standard deviations, are summarized in Table-4.

The largest ΔTP value was observed for Ultra Bond cement at 150 µm (0.63 ± 0.00), indicating the greatest reduction in translucency. The smallest ΔTP value was observed for Panavia V5 at 50 µm (0.30 ± 0.01), indicating minimal change. The Kruskal-Wallis test indicated a significant difference among the groups, confirming that the type of resin cement significantly affects translucency changes (P<0.001). However, the Bonferroni post hoc test showed no significant differences among cements of the same thickness between light-cure and dual-cure types.

So, light-cure cements tended to exhibit slightly greater translucency changes compared to dual-cure cements.

### Assessment of Thickness Changes

Calculating the Spearman correlation coefficient showed that there was a relative and direct correlation between the thickness and discoloration (r=0.425 and P<0.001) and the ΔE value increases with increasing thickness. There was also a direct correlation between the thickness and the translucency changes (r=0.696 and P<0.001) and the ΔTP value increases with increasing thickness (Figure-2 and -3).

## Discussion

In this study, we evaluated the effects of resin cement type and thickness on color stability and cement translucency. Based on previous research, the CIE Lab system is widely used to assess dental material discoloration due to its reliable and reproducible results. However, there are inconsistencies in the literature regarding ΔE values, which indicate the threshold at which color changes are perceptible to the human eye and clinically significant. In simillar studies, Stober et al. [19] considered total discoloration (ΔE) of 2-3 as noticeable, while Seghi et al. [20] suggested ΔE=1 as distinguishable. Kuehni and Marcus [21] reported that only about 50% of participants could detect ΔE=1, whereas Chang J et al. [22] noted that the human eye generally cannot detect ΔE≤1, and only trained observers can identify changes between 1 and 3.3. In our study, consistent with most previous studies [14][16][17][22], color changes below ΔE=3.3 were considered clinically acceptable, while values above were deemed unacceptable. Color changes in resin cements under clinical conditions can occur due to external or internal factors. External factors, such as colored beverages, foods, and cigarette smoke, induce cement discoloration over time due to direct contact with the oral environment, particularly at restoration margins. In contrast, internal factors, including chemical composition, filler type, resin matrix structure, polymerization method, type of initiator, and C=C bond ratio, also significantly influence color stability [1][6][7][8][9][10].

Previous studies assessing external factors often employed immersion of samples in colored solutions. For example, Shiozawa M [2] and Bayindi F [23] immersed resin cement samples in coffee for 24 hours, while Malekipour et al. [24] examined discoloration after 14-day immersion in common beverages such as tea, coffee, lemonade, and soft drinks. Conversely, to study internal factors, different aging protocols have been applied, with thermocycling according to ISO standards being the most common, as it effectively evaluates physical, chemical, and visual changes in non-metallic materials such as composites and dental cements [18].

Our results indicated that both amine-free dual-cure and light-cure resin cements exhibited clinically acceptable discoloration (ΔE ≤ 3.3), but light-cure cements demonstrated superior color stability, consistent with previous studies [1][11]. This difference highlights that polymerization initiator type and chemical composition directly affect long-term discoloration. Several factors, resin matrix, initiator concentration and type, oxidation of unreacted monomers, filler volume, and pigment content, have been shown to influence resin cement color stability [25][26][27][28].

Regarding the resin matrix, Khokhar ZA et al. [29] reported that UDMA is more resistant to color change than BIS-GMA, and Pearson GJ et al. [30] found that UDMA-containing resins absorbed water but exhibited less discoloration than BIS-GMA resins under normal curing conditions. Filler type also affects staining susceptibility, with agglomerated nanoparticles or nanoclusters showing higher discoloration [31][32]. Manojlovic D et al. [33] observed that TPO-containing and FIT-based resin composites had better color stability than CQ- and BisGMA-based composites, despite similar baseline colors.

Building on these findings, amine-reduced light-cure cements (e.g., Variolink NLC Clear) demonstrated the lowest ΔE after thermocycling [11]. Our study, using new amine-free dual-cure and benzoyl peroxide cements (NX3, Panavia V5), confirmed that both dual-cure cements maintained clinically acceptable color stability, aligning with previous results [1][11].

A novel aspect of our study was the evaluation of color stability across different cement thicknesses. Increasing the cement thickness from 50 μm to 150 μm increased ΔE values, although all remained clinically acceptable (ΔE ≤ 3.3). Notably, thicknesses above 150 μm may reduce bond strength and increase failure rates in resin cements [6].

Recent studies calculate the Translucency Parameter (TP) index to assess resin cement translucency, although no consensus exists on clinically acceptable TP thresholds [23][24]. Ghavam et al. [16] found that aging increased opacity in both light-cure and dual-cure cements, which partially aligns with our results. However, unlike our study, they reported that dual-cure cements showed the highest increase in opacity, whereas we observed less translucency change in new amine-free dual-cure cements compared to light-cure cements, likely due to their unique chemical composition involving amine-free and benzoyl peroxide initiators.

Finally, it is important to note that in vitro aging methods cannot fully replicate oral conditions, and the effects of other aging protocols, such as UV radiation, were not assessed in this study. Inclusion of such methods could further enhance the simulation of clinical conditions and provide a more comprehensive understanding of color stability and translucency changes.

## Conclusion

Finally, the evaluation of color stability and translucency under a combination of different aging methods is suggested in future studies, given that aesthetic restorations are exposed to light and UV radiation in the anterior region. With regard to the effect of discoloration and translucency of cements on the final appearance of ceramic restorations, future research can investigate the effect of new amine-free dual-cure color changes and cement translucency on the final appearance of ceramic restorations.

## Conflict of Interest

None.
